# Identifying cases of heroin toxicity where 6-acetylmorphine (6-AM) is not detected by toxicological analyses

**DOI:** 10.1007/s12024-016-9780-2

**Published:** 2016-04-25

**Authors:** Ashley D. Ellis, Gerald McGwin, Gregory G. Davis, Daniel W. Dye

**Affiliations:** 1 Virginia Commonwealth University School of Medicine, 1101 E. Marshall Street, PO Box 980662, Richmond, VA 23298 USA; 2 University of Alabama at Birmingham, 1515 6th Ave. S, Birmingham, AL 35233 USA

**Keywords:** Forensic pathology, Forensic toxicology, Heroin toxicity, Morphine toxicity, Codeine toxicity, Death certification, Drug abuse

## Abstract

**Purpose:**

Heroin has a half-life of 2–6 min and is metabolized too quickly to be detected in autopsy samples. The presence of 6-acetylmophine (6-AM) in urine, blood, or other samples is convincing evidence of heroin use by a decedent, but 6-AM itself has a half-life of 6–25 min before it is hydrolyzed to morphine, so 6-AM may not be present in sufficient concentration to detect in postmortem samples. Codeine is often present in heroin preparations as an impurity and is not a metabolite of heroin. Studies report that a ratio of morphine to codeine greater than one indicates heroin use. We hypothesize that the ratio of morphine to codeine in our decedents abusing drugs intravenously will be no different in individuals with 6-AM present than in individuals where no 6-AM is detected, and we report our study of this hypothesis.

**Methods:**

All accidental deaths investigated by the Jefferson County Coroner/Medical Examiner Office from 2010 to 2013 with morphine detected in blood samples collected at autopsy were reviewed. Five deaths where trauma caused or contributed to death were excluded from the review. The presence or absence of 6-AM and the concentrations of morphine and codeine were recorded for each case. The ratio of morphine to codeine was calculated for all decedents. Any individual in whom no morphine or codeine was detected in a postmortem sample was excluded from further study. Absence or presence of drug paraphernalia or evidence of intravascular (IV) drug use was documented in each case to identify IV drug users. The proportion of the IV drug users with and without 6-AM present in a postmortem sample was compared to the M/C ratio for the individuals.

**Results:**

Of the 230 deaths included in the analysis, 103 IV drug users with quantifiable morphine and codeine in a postmortem sample were identified allowing for calculation of an M/C ratio. In these IV drug users, the M/C ratio was greater than 1 in 98 % of decedents. When controlling for the absence or presence of 6-AM there was no statistically significant difference in the proportion of IV drug users when compared to non IV drug users with an M/C ratio of greater than 1 (*p* = 1.000).

**Conclusion:**

The M/C ratio in IV drug users, if greater than 1, is seen in deaths due to heroin toxicity where 6-AM is detected in a postmortem sample. This study provides evidence that a M/C ratio greater than one in an IV drug user is evidence of a death due to heroin toxicity even if 6-AM is not detected in the blood. Using the M/C ratio, in addition to scene and autopsy findings, provides sufficient evidence to show heroin is the source of the morphine and codeine. Listing heroin as a cause or contributing factor in deaths with evidence of IV drug abuse and where the M/C ratio exceeds 1 will improve identification of heroin fatalities, which will allow better allocation of resources for public health initiatives.

## Introduction

Because heroin is rapidly deacetylated in whole blood, the presence of 6-acetylmorphine (6-AM) in postmortem samples of urine or blood is often used to identify heroin use by a decedent [1, 2]. However, 6-AM has a half-life of 6–25 min before it is metabolized to morphine in the liver [2]. Furthermore, codeine is often present in heroin as an impurity, and not produced by metabolism of the drug. Typically, only small amounts of codeine are detected in samples and researchers have used a morphine to codeine ratio greater than one and the presence of 6-AM in a postmortem sample to identify cases of heroin use [3–5]. Because of the rapid metabolization of heroin and 6-AM, morphine and codeine may be the only substances detected in postmortem samples in cases of heroin use [3–5]. To confirm that cases are being properly classified as deaths due to heroin toxicity, we examined the ratio of morphine to codeine in decedents with and without evidence of intravenous drug use and correlated the ratios with the absence or presence of 6-AM in a postmortem samples. We hypothesize that there will be no difference in the proportion of cases in which the blood morphine to codeine ratio exceeds one in IV drug users with 6-AM detected in a postmortem sample when compared to IV drug users without 6-AM detected in a postmortem sample. If this hypothesis is correct then it has important implications for death certification in that it would be appropriate to classify a death as heroin toxicity with a blood morphine to codeine ratio exceeding one in a setting that suggests IV drug use even in the absence of 6-acetylmorphine. We report here the results of this analysis.

## Methods

All unnatural or suspicious deaths occurring in Jefferson County, Alabama are investigated by the Jefferson County Coroner/Medical Examiner Office (JCCME). Samples of blood, urine, vitreous humor, liver tissue, and brain tissue are routinely collected (if possible) in all cases and submitted for toxicological analyses for drugs of abuse. The analyses are performed by the Forensic Toxicology Laboratory at the University of Alabama at Birmingham by contract with the JCCME. Toxicology reports indicate the absence or presence of 6-AM in screening tests of urine and blood. Notably, 6-acetylmorphine is routinely tested for in urine samples (when urine is available) using Enzyme Multiplied Immunoassay Technique (EMIT). If no urine is available for testing, a blood sample is tested using EMIT. Additional testing of other samples for 6-acetylmorphine is not routinely performed and only samples of urine or blood were screened during the time period of selected cases. If 6-AM or opiates are detected by EMIT then additional testing is performed to confirm the presence of morphine, codeine, and 6-AM (if present) in samples of blood with quantitation. In most cases a positive 6-AM was from a sample of urine; however, for purposes of the analysis, a positive blood or urine 6-AM are considered unequivocal. Information from the toxicology report and evidence of intravascular (IV) drug use is collected in the JCCME case management database for each case. A search of the database for accidental deaths in 2010–2013 in which morphine was detected in a postmortem sample was performed. From these cases only deaths due to drug toxicity (drug overdose deaths) were included in the study. Cases where trauma was listed as the cause of death and drug use was listed as a contributory cause of death (motor vehicle fatalities) were also excluded. In every case, toxicology reports were reviewed and the concentrations of morphine and codeine were documented. If codeine was not detected in a case, a result of “not detected” was recorded. The absence or presence of 6-AM was also recorded for each case. In every case, the files were reviewed to determine if the decedent was an IV drug user. Criteria for inclusion as an IV drug user included the presence of needles or syringes at the scene, a reported history of IV drug use to investigators, identification of a recent needle puncture mark on the body not related to medical attention, needle track marks identified on the body, or polarizable foreign body material in pulmonary lymphovascular spaces identified during microscopic examination.

From the collected data, a morphine to codeine ratio (M/C ratio) was calculated in every case that had a quantifiable amount of morphine and codeine in a postmortem sample [4]. From decedents with a quantifiable M/C ratio, the proportion of cases in which the M/C ratio was greater than one was compared between IV drug users and non-IV drug users. In addition, the proportion of cases in which the M/C ratio was greater than one was compared between IV drug users with 6-AM present or absent in a sample. Using this information, an algorithm for interpreting toxicological results in cases with morphine and codeine detected in a postmortem sample was designed.

## Results

We identified 190 deaths due to drug toxicity. From this sample, 110 decedents had 6-AM detected in a postmortem sample. Of these cases, 108 (98 %) had a morphine to codeine ratio of greater than one as would be expected in decedents using heroin. From the original 190 cases, 127 IV drug users were identified, of whom 103 had quantifiable morphine and codeine detected in a postmortem sample. The postmortem sample was always a blood sample and most commonly was blood collected from the internal iliac veins at autopsy. All but two (98 %) of the 103 decedents had an M/C ratio of greater than one. Among the 63 non-IV drug users with quantifiable morphine and codeine detected, all but one (98 %) had an M/C ratio of greater than one (Table 1). Compared to all IV drug users this difference was not statistically significant (*p* = 1.00). Of the 103 IV drug users with quantifiable morphine and codeine in a postmortem sample, 69 decedents (68 %) had 6-AM detected in a postmortem sample and 34 (33 %) had no 6-AM detected in a postmortem sample. When compared to the 63 non-IV drug users with quantifiable morphine and codeine detected, 41 decedents had 6-AM detected in a postmortem sample (*p* = 0.87).

**Table 1:** Comparison of IV and non-IV drug users

	Total	M and C present in sample^a^	6-AM present^b^	M/C > 1	Morphine range	Codeine range
IV drug user	127	103	69	101	Min: 0.130 mg/L Max: 1.48 mg/L	Min: 0.002 mg/L Max: 0.180 mg/L
Non-IV drug user	63	63	41	62	Min: 0.016 mg/L Max: 1.70 mg/L	Min: 0.002 mg/L Max: 0.042 mg/L

^a^Both morphine and codeine must be present in quantifiable concentrations

^b^6-AM present in urine or blood by EMIT

A total of 166 cases with quantifiable morphine and codeine in a postmortem sample were also analyzed. From these cases 163 deaths had an M/C ratio of greater than one. From the 163 cases, 108 had 6-AM detected in a postmortem sample. Of the remaining 55 decedents, 6-AM was not detected in a postmortem sample; however, 33 of the decedents were IV drug users (Table 2). Our study has found no statistically significant difference between individuals with a history of intravenous drug abuse and individuals with no known history of intravenous drug abuse with respect to either M/C ratio >1 or the presence of 6-AM. Therefore, we conclude that M/C > 1 in an IV drug user is sufficient evidence to infer heroin use by a decedent even if 6-AM is not detected in a postmortem sample. The only exception to making a diagnosis of heroin use by such inference would be the presence of various medications that contain morphine sulfate and codeine at the scene of death.

**Table 2:** Comparison of all cases with quantifiable Morphine and Codeine

	Total	6-AM present^a^	IV drug use positive^b^
M/C > 1	163	108	33
M/C < 1	3	2	0

^a^6-AM present in urine or blood by EMIT

^b^IV drug users with no 6-AM detected in a postmortem sample

These data allowed for classification of deaths as “heroin toxicity” when an M/C ratio exceeded one, evidence of IV drug use was identified by investigation or postmortem examination, and no 6-AM was detected in a postmortem sample.

## Discussion

Based on data from the CDC approximately 43,000 accidental deaths due to drug toxicity were reported on death certificates in 2013 [6]. Other studies have reported concern for underreporting of deaths due to heroin use because the inability to identify 6-AM in a postmortem sample, leads to classification of these deaths as being due to morphine use [3, 7, 8]. These studies suggest that heroin toxicity may be responsible for overestimating the contribution of prescription opiates to accidental deaths in the United States [3, 7, 8]. Other studies have suggested that codeine is also over reported as a cause of unintentional drug overdose deaths for similar reasons [9, 10].

**Fig. 1:**
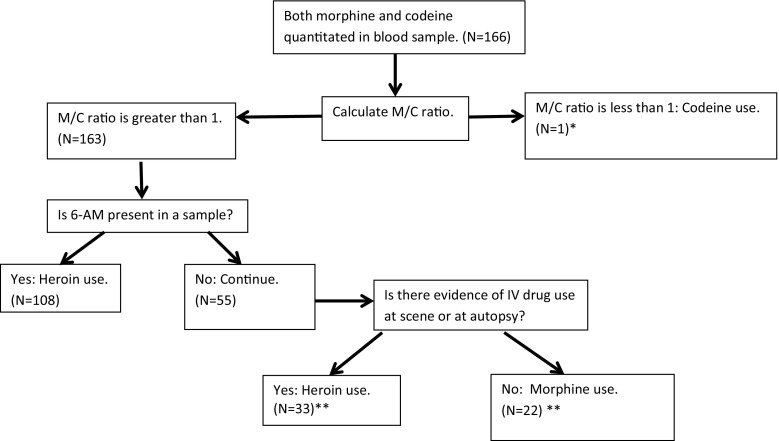
Interpreting morphine and codeine detected by toxicological testing: *single asterisk* Two other cases in our study had an M/C ratio of less than one; however, 6-AM was present in a sample. These cases were classified as “heroin use” as 6-AM will override an M/C ratio of less than 1. *Double asterisk* an exception to making a diagnosis of heroin use by such inference would be allowed if the presence of various medications that contain morphine sulfate and codeine were found at the scene of death

When interpreting toxicological results from a postmortem sample, forensic pathologists must not only consider what drugs are detected, but also drug concentrations, and ratios [5, 11, 12]. The ratio of morphine to codeine is of paramount importance as morphine is a metabolite of heroin and codeine, and detection of morphine could indicate the use of either drug or use of morphine sulfate [4, 5, 12–14]. A morphine to codeine ratio of less than one suggests use of codeine, and a morphine to codeine ratio of greater than one suggest heroin use or use of morphine sulfate [4, 5, 12, 13]. Codeine is an alkaloid prepared from opium poppy (*Papaver somniferum*) by methylation. Heroin produced from opium often contains small amounts of codeine as an impurity [15, 16]. Some research suggests that some commercial morphine sulfate preparations may also contain codeine, while other researchers have suggested that codeine is a minor metabolite of morphine sulfate in humans [14–16]. When a morphine to codeine ratio of greater than one is encountered, additional toxicological testing for 6-AM may be all that is needed to discern a case of heroin use [1]. Furthermore, 6-AM positivity in urine or blood can outweigh an M/C ratio of less than one as it did in two cases. These cases were properly classified as deaths due to heroin use. However, if 6-AM cannot be detected, and the M/C ratio is greater than one, then the absence or presence of evidence of IV drug use should be considered. If an individual has a morphine to codeine ratio of greater than one and evidence of IV drug use is identified by investigation (needles/syringes/spoon at scene) or at autopsy (needle track marks/recent needle puncture mark not placed during medical intervention/polarizable material in pulmonary lymphovascular spaces); an overdose death can be classified as heroin toxicity without identifying 6-AM in a postmortem sample. The Jefferson County Coroner/Medical Examiner Office uses an algorithm for classification of deaths where both morphine and codeine are detected in a postmortem sample (Fig. 1).

Because national surveillance of opiate related fatalities is based on death certificate data, proper classification of deaths is imperative. Testing of additional samples for 6-AM may help to discern deaths due to heroin; however, information from scene investigations or the postmortem examination identifying a decedent as an IV drug user can identify a death due to heroin toxicity when 6-AM is not detected in a postmortem sample. We suggest the classification of heroin toxicity, morphine toxicity, or codeine toxicity using the algorithm listed above after consideration of the findings at autopsy, interpretation of postmortem toxicological testing, and correlation of scene investigations. If cases do not fit the algorithm or conflicting evidence at the scene does not correlate with the toxicological results, a classification of “opiate toxicity” may be applied; however, after using the classification algorithm for 3 years, one of the authors (GGD) has not received any complaints from family members, law enforcement, or the Alabama Department of Heath questioning the validity of ascribing death to the effects of heroin on the Death Certificate in those cases. Furthermore, scene investigations haven’t discovered medications containing morphine and codeine at scenes in cases with an M/C ratio of greater than 1 and no 6-AM detected in a postmortem sample. As we alluded in the algorithm, such a possibility exists; however, in our experience such a scenario has not occurred.

We do not claim that the algorithm we recommend is infallible, but infallibility is not a requirement for death certification. The cause of death is a diagnosis, which is an opinion. As forensic pathologists we regularly ascribe death to the effects of coronary artery atherosclerosis, even when we have no evidence for myocardial infarction beyond sudden, unexpected death and at least one coronary artery significantly narrowed by plaque and no other anatomic or toxicological cause for death. Clinicians sometimes find this practice irresponsible, claiming that without ECG changes or elevated biochemical markers we cannot make a determination that coronary artery disease caused death. The algorithm that we recommend for classification of these selected cases is analogous. By using this algorithm a forensic pathologist who ascribes death to heroin toxicity will be correct in the vast majority of cases, and in doing so forensic pathologists will provide the public health system a far more accurate determination of the contribution of heroin to death. Proper classification of these deaths will allow for better guidance of public health strategies and action plans to enhance medical rehabilitation programs and prevent these deaths.

## Key points

1. Deaths due to heroin toxicity can be identified even if no 6-acetylmorphine can be detected in samples of urine or blood collected at autopsy.
2. When both morphine and codeine are detected and quantified in a postmortem sample of blood in a death due to an apparent intoxication a morphine to codeine ratio (M/C ratio) can be calculated. In these cases, presence or absence of evidence of intravascular drug use should also be considered.
3. Our study has found no statistically significant difference between individuals with a history of intravenous drug abuse and individuals with no known history of intravenous drug abuse with respect to either M/C ratio >1 or the presence of 6-AM.
4. If the M/C ratio is greater than one, and there is evidence that the decedent is an intravascular drug user, then a death due to heroin use can be identified in cases where 6-AM was not detected in a postmortem sample.
